# Management of left atrial myxoma in pregnant women: a case series

**DOI:** 10.1186/s13019-024-02747-2

**Published:** 2024-04-10

**Authors:** Yanli Liu, Haiping Wang, Huanlei Huang, Fengzhen Han, Jian Zhuang, Yanqiu Ou, Yanyan Lin, Weina Zhang

**Affiliations:** 1 Department of Obstetrics, Guangdong Provincial People’s Hospital(Guangdong Academy of Medical Sciences), Southern Medical University, No. 106 Zhongshan 2nd road, Guangzhou, 510080 Guangdong China; 2 Prenatal Diagnosis Center, Guangdong Provincial People’s Hospital(Guangdong Academy of Medical Sciences), Southern Medical University, No. 106 Zhongshan 2nd road, Guangzhou, 510080 Guangdong China; 3 Department of Cardiac Surgery, Guangdong Cardiovascular Institute, Guangdong Provincial People’s Hospital(Guangdong Academy of Medical Sciences), Southern Medical University, No. 106 Zhongshan 2nd road, Guangzhou, 510080 Guangdong China

**Keywords:** Cardiac myxoma, Pregnancy, Cardiopulmonary bypass, Totally endoscopic minimally invasive cardiac surgery, Outcome

## Abstract

**Background:**

Left atrial myxoma during pregnancy is rare. We present three cases in order to aid in the management.

**Case Presentation:**

Three cases of left atrial myxoma during pregnancy were presented in this article. Three patients all received multidisciplinary team work and acquired good outcomes. The case 1 had no symptoms and delivered before traditional cardiac surgery. The case 2 and case 3 undergone totally endoscopic minimally invasive cardiac surgery during pregnancy. The case 3 maintained pregnancy to term and gave birth to a healthy baby via vaginal delivery. No relapse of the tumor was observed.

**Conclusions:**

The management of left atrial myxoma during pregnancy ought to be individualized and combined with the gestational age. If the diagnosis was made in the first two trimesters of pregnancy, totally endoscopic minimally invasive cardiac surgery during pregnancy would be an optimal choice. The patients can benefit from the multidisciplinary team work.

## Background

Cardiac myxomas are the most common primary benign tumors of the heart, accounting for 30–50% [1]. Myxomas are predominant in females, with 65% of myxomas occurring in women between the third and sixth decade of life [2]. The vast majority of myxomas (75%) occur in the left atrium [2]. Only about 57 cases of myxomas in pregnancies have been reported in the literature. To aid in the management of cardiac myxoma during pregnancy, we present three cases of left cardiac myxomas occurring in pregnancy and review some literature.

## Case presentation

### Case 1

A 27-year-old primigravida woman was initially evaluated at a gestational age of 12 weeks in a local hospital. Transthoracic echocardiography (TTE) was performed as part of a routine examination and revealed a 32 × 26 mm mass in the left atrium with an appearance suggestive of atrial myxoma. She was asymptomatic and refused the cardiac surgery during pregnancy.

When entered into the third trimester of pregnancy, she still had no abnormal complaints and all of her obstetrical examinations, especially the fetal development, were normal. At 33 + weeks’ gestation, she was transferred to our hospital and denied any shortness of breath, fatigability, chest discomfort, palpitations, light headedness, or embolic symptoms. Her past medical history was normal. On physical examination, her cardiac rhythm was regular. She was observed to have a mild systolic murmur. No gallop, “tumor plop”, or click was audible.

**Fig. 1:**
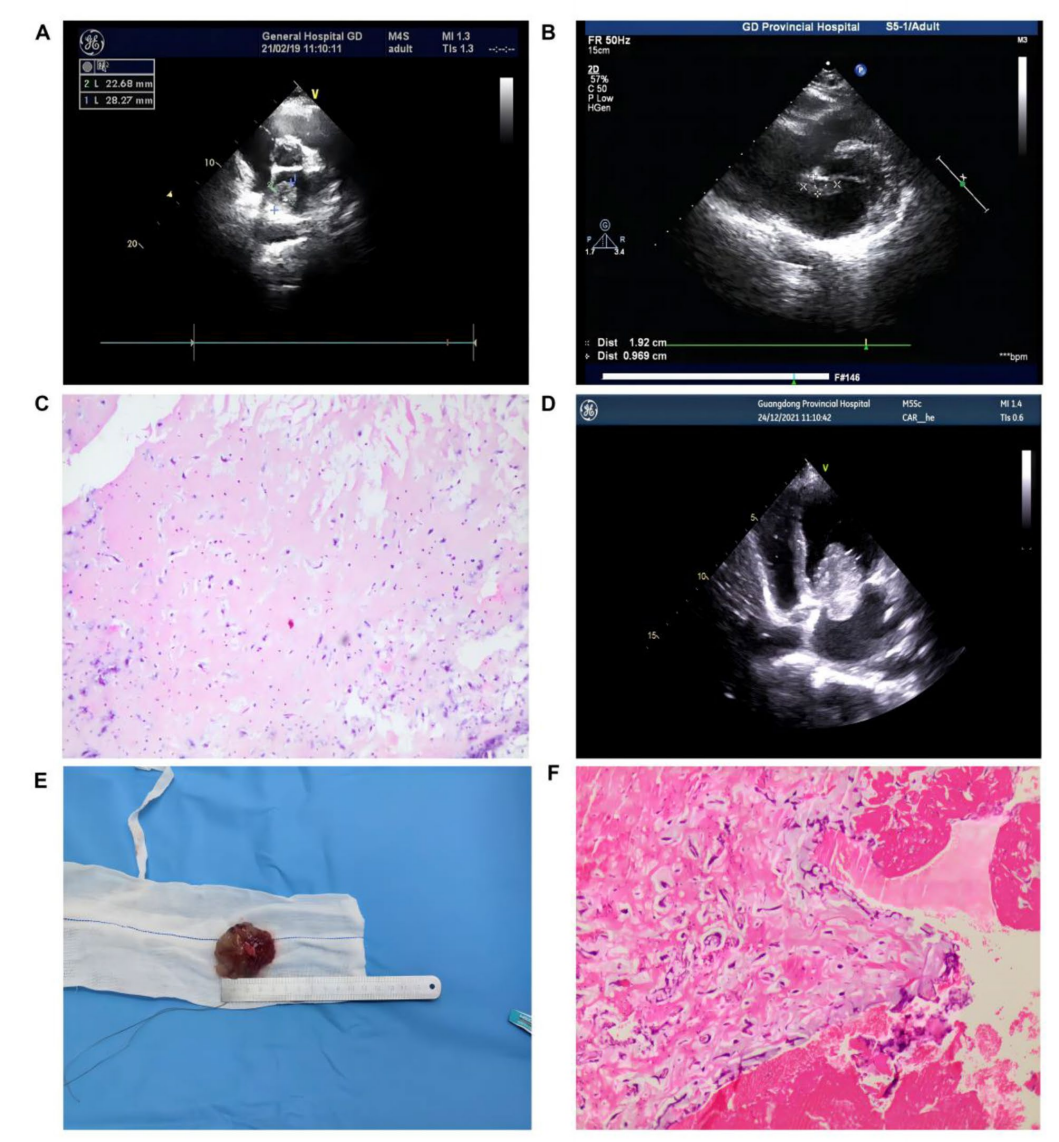
Representative images of the patients. **A**. TTE image of case 1: in the parasternal short-axis great arteries view, the left atrial myxoma (28.27 × 22.68 mm) adherent to the posterior wall; **B**. TTE image of case 2: in the non-standard view, a mobile, pedunculated 19 × 10 mm mass in the left atrium attached to the fossa ovalis; **C**. Histological examination confirms the diagnosis of cardiac myxoma in case 2; **D**. TTE image of case 3: in the apical four-chamber view, a mobile, pedunculated 45 × 33 mm mass in the left atrium attached to the interatrial septum; **E**. Appearance of left atrial myxoma in case 3; **F**. Pathological image of case 3

TTE showed a 28.27 × 22.68 mm pedunculated mass in the left atrium, adherent to the posterior wall (Fig. 1A)and accompanied by mild mitral regurgitation (MR). There was no evidence of ventricular inflow obstruction. Laboratory values and electrocardiogram(ECG) results were normal. We convened a multidisciplinary team composed of obstetrician, cardiologist, surgeon and anesthetist to discuss the management. Since a fetus of this gestational age could survive after birth with a low risk of premature complications and surgery under cardiopulmonary bypass (CPB) during pregnancy may carry a risk of fetal death in the uterus, we finally chose to deliver the fetus first. The patient delivered a preterm infant at 35 weeks of gestation via Cesarean section. The infant had a normal appearance and weighed 2770 g with an Apgar score of 10. Five months after delivery, she underwent a traditional cardiac surgery, and the tumor was removed. Thus far, the patient feels well, and there is no evidence of a relapse.

### Case 2

A 32-year-old gravida 5, para 2 woman was referred to our hospital at 17 weeks’ gestation with symptoms of palpitations and shortness of breath. TTE showed a mobile, pedunculated 19 × 10 mm mass in the left atrium (Fig. 1B) attached to the fossa ovalis. No mitral regurgitation was revealed. The cardiac rhythm was regular and no murmurs were heard in any of the valve regions of the heart. Her lungs were clear. No symptoms of peripheral embolization were found. The blood tests did not show any positive results. ECG revealed an incomplete right bundle-branch block.

After cardiovascular, surgical and obstetrical consultations, surgical excision was recommended. At 18 weeks’ gestation, the patient experienced a removal of the tumor via totally endoscopic minimally invasive cardiac surgery. CPB was continued for 46 min, and the duration of aortic cross clamp was 21 min. Normothermia was applied to the patient during CPB. A 20 × 15 mm mass was eliminated from the left atrium. Pathological examination indicated a myxoma(Fig. 1C). TTE suggested that there were no residual mass, either during or after the operation.

Both the mother and the fetus tolerated the operative procedure, but the patient developed uterine contractions two days after the procedure. We chose to observe, and the contractions subsided. The patient recovered without any complications. However, the fetus was confirmed to have a chromosome abnormality (45,XO chimerism )at 24 weeks’ gestation. The woman chose a termination of the pregnancy at 26 weeks’ gestation. Two years later, the patient got pregnant again and gave birth to a healthy baby uneventfully. So far, there is no evidence of a relapse.

### Case 3

A 27-year-old primigravida woman presented for a routine obstetrical examination at 19 weeks’ gestation without any symptoms. TTE revealed a mobile, pedunculated 45 × 33 mm mass in the left atrium (Fig. 1D) attached to the interatrial septum, with an appearance suggestive of atrial myxoma. The mass was observed to have prolapsed through the mitral valve into the left ventricle. However, it was not accompanied by mitral regurgitation. There was an obvious “tumor plop” on physical examination. The patient sometimes felt fatigued. There was no evidence of embolization. Laboratory tests and ECG did not show any alteration from normal values.

After multidisciplinary team evaluation, we believed that the surgical removal of the tumor in the second trimester of pregnancy would benefit to the patient, because the tumor was more bigger than the cases before and carried a high risk of complete obstruction of the mitral valve and embolization. The patient finally chose the cardiac surgery during pregnancy. At 20 weeks’ gestation, we performed totally endoscopic minimally invasive cardiac surgery on the patient and took off the entire mass. CPB lasted for 68 min, and the duration of aortic cross clamp was reduced to 49 min. The patient’s temperature was kept normal during CPB. We incised the right atrium and entered the left atrium by excising the fossa ovalis. A large smooth pedunculated mass with typical external appearance of a myxoma was encountered and excised with its stalk from the interatrial septum (Fig. 1E). The diagnosis of myxoma was confirmed by pathological evaluation(Fig. 1F). Postoperative transesophageal echocardiography(TEE) revealed no residual mass.

The patient developed moderate uterine contractions related to the CPB after the operation and was treated with atosiban (a contraction inhibitor without adverse cardiovascular effects). The contractions later subsided. Her fetus survived, and no abnormalities were confirmed by ultrasound. She underwent normal spontaneous vaginal delivery of a healthy boy infant at 37 weeks’ gestation. The infant weighed 2590 g, and the Apgar score was 10. The mother and her infant are followed up every month, and no abnormal findings have been detected at present. No relapse of the tumor was observed.

## Discussion and conclusions

Primary tumors of the heart are uncommon, with an estimated incidence of 0.17–0.19% in selected autopsy series. Of these tumors, 75% are benign, and approximately 50% of these are myxomas [3]. A myxoma is a neoplasm of unknown histogenesis that occurs most often in middle-aged, usually female patients, who are readily amenable to treatment and have an excellent postoperative prognosis [4]. Myxomas usually occur as a single lesion in the left atrium [5]. Over 90% of myxomas are located in the atria, with up to 83% located in the left atrium, as found in the three cases presented above, and 13% located in the right atrium [1, 2] .

Left atrial myxoma in pregnant patients is extremely rare. Fifty-one cases were reported in the most recent literature review in 2015 [6], and another 6 cases were reported later [7–12]. As the Guangdong provincial obstetrical cardiology intensive care center in China, our hospital has accumulated a significant amount of clinical data on pregnant women with heart disease, and three cases of left atrial myxomas presented above were found by searching a recent 10-year clinical database. All the patients achieved good outcomes via multidisciplinary team work.

Clinical features lie on the site, size, mobility, and surface stigmas of the myxoma. Typically, patients could exhibit cardiac, embolic or nonspecific systemic symptoms, which consist of fever, loss of weight, exhaustion and myalgia [13]. Two of the previous cases manifested the signs of stroke, and one patient carrying a right heart myxoma showed characteristics of pulmonary embolism [14]. In our cases, the presenting symptoms involved palpitation, dyspnea and fatigability. The patients had no complaints of chest pain, dizziness, light headedness or other embolic symptoms, unlike those reported in the previous literature. Surprisingly, the patient in case 1 showed no symptoms during the entire pregnancy, the same as the senile patient that Tetera et al [15] reported in 2022, which could be explained by the the presence of immobile, non-obstructive, and well-circumscribed myxomas. On the other hand, we should exclude other asymptomatic primary cardiac tumors such as lipomas, which are 50 times less common than myxoma and grow very slowly. They usually arise from the epicardial fat tissue, growing into the pericardial sac. The most frequent intracardiac location is the right atrium, seen in report from Fabrizio [16]. The TTE examination can assist to exclude the diagnosis of a lipoma.

Generally, surgical resection of myxoma is recommended for the non-pregnant women; nonetheless, for pregnant women, the management becomes complicated, because we should weight the potential risk of both mother and fetus, such as the adverse effects on fetus, severe complications of preterm birth, and the potential of myxoma embolization for mother [8]. With regard to the rarity of these cases, individualized treatment is thought to be vital. A. S. John et al. indicated that if the tumor manifests the traits of small size and non-mobility and shows no valvular obstruction, the decision of observation may be acceptable. Reversely, if the tumor is too large and leads to inflow block, or is mobile and carries a high risk of embolism, surgical removal of the myxoma during pregnancy is ought to be taken into consideration [5]. Traisrisilp K et al [17] recommended that the surgical therapy rests with the gestational age. If the tumor is found in the first trimester of pregnancy, the operation should be performed early in the second trimester of pregnancy. If the tumor is diagnosed after the first trimester but before the late third trimester, surgery is supposed to be carried out without delay. If the diagnosis takes place in the late third trimester of pregnancy, delivery via cesarean section should be carried out first, in view of the consensus that fetus in this trimester would gain a high probability of survival without severe complications. Our management strategies were the same as those of Traisrisilp. In Case 1, the nature of the left atrial myxoma was immobile and non-obstructive, which may make the patient carry a low risk of sudden death and embolism, just like the report from Tetera et al. [15] that the elderly patient had no symptoms for a long time. Therefore, the patient can maintain the pregnancy to the late third trimester when a fetus could survive with few premature complications. At last, we chose to deliver the fetus first.In both Case 2 and Case 3, the diagnosis was made in the second trimester of pregnancy. Considering the high risk of maternal embolization stemming from the mobile tumor, we performed surgical resection of the tumors during pregnancy. The removal of left atrial myxomas was relatively uncomplicated, due to the simple surgical access and fewer feeding vessels of the tumor, which was quite different from the other cardiac benign tumor. Take the cardiac paraganglioma for example, it may be highly vascularized and closely related to the surrounding structures, which make the surgery more difficult, seen in reports from Fabrizio [18] and González López [19]. Furthermore, the CPB duration in left atrial myxomas was much shorter than other cardiac tumors. Although CPB during the cardiac operation would result in a certain probability of fetus death in the uterus, the maternal and fetal outcomes would improve if CPB was appropriately applied, such as a high flow rate, high perfusion pressure and pulsatile flow. Moreover, the short CPB time can also improve the fetal survival rate. Fortunately, the mothers and fetuses tolerated the operation.

Nevertheless, some researchers [6] suggest that cardiac operation should be taken into consideration in view of the adverse effects on the pregnant women, especially the embolism complications, no matter what gestational age is. It has been reported that the maternal survival rate has reached to 100% in the pregnant women with a cardiac myxoma, much more higher than that of the pregnant patients with infective endocarditis [20]. The fetal outcome would be promoted if suitable CPB skills were performed in cardiac operation during pregnancy, containing a high flow rate, high perfusion pressure, pulsatile flow, and normothermia [6]. Liu et al. [21] have reported 22 cases of women accepting cardiac surgeries under CPB during pregnancy, in which the maternal mortality was 0%, and the fetal mortality was 18.2%, much lower than that of previous reports. Therefore, the surgical resection of left atrial myxoma during pregnancy could be an acceptable option if CPB is appropriately used. In the present literature, the minimum gestational age was 6 + weeks [3, 22], and the fetal outcomes were good. In our cases, two patients received totally endoscopic minimally invasive cardiac surgery during the second trimester of pregnancy under CPB. None of the mothers and fetuses experienced any adverse complications. Totally endoscopic minimally invasive cardiac surgery has the advantages of small trauma, beautiful incision and rapid postoperative recovery [23]. In case 2 and case 3, the patients both accepted the minimally invasive cardiac surgery and acquired better recovery and shorter duration of hospital stay(3 days) than the traditional surgery(the median sternotomy), indicating that the minimally invasive cardiac surgery is also suitable to the pregnant woman.

It has been recommended by guidelines [24, 25] that a multidisciplinary team or pregnancy heart team should be invited to the management of pregnant women with cardiovascular disease. The minimum team must involve a cardiologist, obstetrician, and anaesthetist, all with expertise in the management of high-risk pregnancies in women with cardiovascular disease [25]. The multidisciplinary team work could greatly improve the effectiveness of treating the high-risk patients. In case 1, we conducted a multidisciplinary team composed of a cardiologist, obstetrician, neonatologist, and anaesthetist. And in case 2 and 3, the pregnancy heart team included a cardiologist, obstetrician, and anaesthetist. All the patients gained good outcomes, indicating that the multidisciplinary team work is beneficial to the pregnant women with left atrial myxoma.

In conclusion, the management of women with left atrial myxomas diagnosed during pregnancy should be individualized and combined with the gestational age. If the diagnosis was made in the first two trimesters of pregnancy, totally endoscopic minimally invasive cardiac surgery would be an optimal choice. CPB can be performed with relative safety for the mother and fetus when adhering to recommended guidelines. The decision must be made by a multidisciplinary team consisting of an obstetrician, cardiologist, anesthesiologist and neonatologist.

## Data Availability

The case data and figures have already been presented in the article. All data related to this report are available from the corresponding author on reasonable request.

## Abbreviations

TTE: Transthoracic echocardiography
MR: Mitral regurgitation
ECG: Electrocardiogram
CPB: Cardiopulmonary bypass
TEE: Transesophageal echocardiography

## References

[1] McManus B, Lee CH, Libby P (2008). Primary tumors of the heart. Brunwald’s Heart Disease.

[2] Reynen K (1995). Cardiac myxomas. N Engl J Med.

[3] Koukis I, Velissaris T, Pandian A (2013). Left atrial myxoma associated with mitral valve pathology in pregnancy. Hell J Cardiol.

[4] Pinede L, Duhaut P, Loire R (2001). Clinical presentation of left atrial cardiac myxoma. A series of 112 consecutive cases.Medicine. (Baltimore).

[5] John AS, Connolly HM, Schaff HV, Klarich K (2012). Management of cardiac myxoma during pregnancy: a case series and review of the literature. INT J CARDIOL.

[6] Yuan SM (2015). Cardiac myxoma in pregnancy: a comprehensive review. Rev Bras Cir Cardiovasc.

[7] Sabzi F, Faraji R (2016). Preoperative Emboli in a pregnant woman with Myxoma. Iran J MED SCI.

[8] Giammarco GD, Marchetti M, Foschi M, Marinelli D, Micucci D, Buca D (2015). Corticosteroid prophylaxis for fetal immaturity in a pregnant patient with atrial myxoma. J Cardiol Cases.

[9] Taksaudom N, Traisrisilp K, Kanjanavanit R. Left Atrial Myxoma in Pregnancy: Management Strategy Using Minimally Invasive Surgical Approach. CASE REP CARDIOL. 2017; 2017:8510160. 10.1155/2017/8510160. 10.1155/2017/8510160 PMC5439244 28567309

[10] Alix J, Pruzansky (2021). Atrial Myxoma presenting pregnancy as NSTEMI in early pregnancy. J AM COLL CARDIOL.

[11] Harrison JHN, Arnolds DE, Banayan JM, Rana S, Schnettler WT, ,Neuburger PJ (2020). Surgical Excision of a left atrial Myxoma during the second trimester of pregnancy. J CARDIOTHOR VASC AN.

[12] Al Riyami N, Nair A, Al Lawati H, Al Kindi AH (2023). Successful management of maternal left atrial myxoma in pregnancy. Oman Med J.

[13] Mochizuki Y, Okamura Y, Iida H (1998). Interleukin-6 and complex cardiac myxoma. ANN THORAC SURG.

[14] Fang YM, Dean R, Figueroa R (2007). Right atrial myxoma mimicking an atrial thrombus in the third trimester of pregnancy. J MATERN- FETAL NEO M.

[15] Tetera W, Wilk A, Król W, Braksator W (2022). Asymptomatic left atrial myxoma. J CARDIOVASC ECHOGR.

[16] Fabrizio Ceresa G, Calarco E, Franzı` (2010). Francesco Patane`. Right atrial lipoma in patient with Cowden syndrome. INTERACT CARDIOV TH.

[17] Traisrisilp K, Kanjanavanit R, Taksaudom N (2017). Huge cardiac myxoma in pregnancy. BMJ Case Rep Published Online.

[18] Fabrizio Ceresa F, Sansone (2010). Mauro Rinaldi and Francesco Patanè. Left atrial paraganglioma: diagnosis and surgical management. INTERACT CARDIOV TH.

[19] González López MT, González SG, García ES (2013). Surgical excision with left atrial reconstruction of a primary functioning retrocardiac paraganglioma. J Cardiothorac Surg.

[20] Yuan SM. Infective endocarditis during pregnancy. JCPSP-J COLL PHYSICI. 2015;25(2):134-9. https://doi.org/02.2015/JCPSP.134139. 25703759

[21] Liu Y, Han F, Zhuang J (2020). Cardiac operation under cardiopulmonary bypass during pregnancy. J Cardiothorac Surg.

[22] Kither HJ, DIS CHILD- FETAL. Atrial myxoma in early pregnancy. ARCH. 2012;97(Suppl1): A52.1-A52. 10.1136/fetalneonatal-2012-301809.166.

[23] Modi P, Rodriguez E, Hargrove WC (2009). Minimally invasive video-assisted mitral valve surgery: a 12-year, 2-center experience in 1178 patients. J THORAC CARDIOV SUR.

[24] Practice Bulletin ACOG 212. Pregnancy and heart disease. OBSTET GYNECOL. 2019;134(4):881–2. 10.1097/AOG.0000000000003497. 10.1097/AOG.0000000000003497 31568352

[25] Regitz-ZagrosekV, Roos-Hesselink JW, Bauersachs J et al. 2018. ESC Guidelines for the. Management of cardiovascular diseases during pregnancy.EUR. HEART J. 2018;39(34):3165–241. 10.1093/eurheartj/ehy340. 10.1093/eurheartj/ehy340 30165544

